# Difficulties in the diagnosis and management of alveolar hydatid disease: A case series

**Published:** 2016

**Authors:** Ghodratollah Maddah, Abbas Abdollahi, Reza Sharifi-Nooghabi, Alireza Tavassoli, Mohammad Taghi Rajabi-Mashadi, Azadeh Jabbari-Nooghabi, Mehdi Jabbari-Nooghabi

**Affiliations:** 1 Endoscopic and Minimally Invasive Surgery Research Center, Ghaem Hospital, Faculty of Medicine, Mashhad University of Medical Sciences, Mashhad, Iran.; 2 Surgical Oncology Research Center, Imam Reza Hospital, Faculty of Medicine, Mashhad University of Medical Sciences, Mashhad, Iran.; 3 Department of Statistics, Faculty of Mathematical Sciences, Ferdowsi University of Mashhad, Mashhad, Iran.

**Keywords:** Alveolar echinoccosis, Echinococcus multilocularis, Diagnosis, Treatment

## Abstract

**Background:**

Alveolar echinococcosis (AE) is a chronic, rare and sometimes lethal parasitic infection in humans, caused by the larval stage of the fox tapeworm Echinococcus multilocularis. This study aimed to investigate the clinical aspects and treatment outcomes of patients with alveolar hydatid disease.

**Methods:**

The medical records of patients with alveolar echinococcosis admitted between 1997 and 2012 were reviewed. Diagnosis was confirmed by physical examination, ultrasonography and CT scanning and MRI. Various treatment techniques were used such as complete liver resection in seven (38.89%) patients, biliary bypass in two (11.11%) patients, laparotomy and tumor biopsy in eight (44.44%) patients and long term medical treatment in one (5.56%) patient. After discharge, all patients were followed to determine the effect of treatment, complications, recurrences and survival.

**Results:**

A total of 18 patients with mean age of 46.11±15.14 years (range 23-74 years) were studied. The disease was more prevalent in women than men (78.9% vs 4, 21.1%, P=0.021). Fourteen (77.78%) patients live in Chenaran, a town located in Khorasan, Iran). Death occurred in (22.22%) patients after an average period of 45.70±7.50 months after disease onset. 14 remaining patients survived after a mean follow-up duration of 54.60±29.17 months.

**Conclusion:**

Diagnosis of alveolar echinococcosis should be considered in endemic area. Early diagnosis and treatment is associated with excellent outcome.

Larval alveolar echinococcosis (AE) also known as alveolar colloid of the liver, is caused by a rodent cestode (Echinococcus multilocularis). Alveolar hydatid disease is a less common disease which is mostly seen in countries with larger reservoirs of hosts (foxes, dogs and wolves) such as Antarctica and Arctic region (1). Biological behavior of larval Echinococcus multilocularis in human is similar to a malignant tumor that is determined by growth of damaging tissues and metastasis to distant organs. The disease has a high mortality rate (more than 90 % within 10 years and virtually 100 % within 15 years of the onset of symptoms) in untreated cases (2). This larva differs from E. granulosus in cystic echinococcosis of the liver. The growth and proliferation of this larva is similar to a slow-growing tumor of the liver and can damage liver function. Sometimes, it is difficult to differentiate it from liver cancer because of invasion to biliary and vascular tissue of the liver. Early diagnosis and radical surgery provide the best chance for definitive treatment and cure (3). Although treatment of AE is less effective than treatment of cystic echinococcus, still the general approach to its treatment remains to be surgery with the purpose of complete resection of infected parts of involved organs. Also, liver transplantation can be a lifesaving approach in patients who are at risk of death (4).

According to our review of literature, alveolar echinococcosis had been reported in Iran as sporadic, (5) but in the past two decades, we detected an endemic area in Chenaran (Khorasan Razavi, Iran) with a population of 108,533 in 26,937 families (6). The aim of this study was to describe the clinical aspects and treatment results in patients with alveolar echinococcosis. 

## Methods

This is a retrospective study of 18 patients presented during a 17 year period between 1997–2012 to Ghaem and Omid Hospitals of Mashhad University of Medical Sciences, Mashhad, Iran. Data were collected by reviewing the medical records. Diagnosis was confirmed by clinical examination and imaging techniques including ultrasonography, CT scanning, and MRI as well as appropriate laboratory tests. In addition, histologic examination and diagnostic laparotomy was also used for definite diagnosis. 

All patients were evaluated by physical examination, ultrasonography and CT scanning of the abdomen. The patients were followed-up for postoperative complications, recurrence and mortality. After discharge, the patients were re-examined for possible complication on the 10th day, 3 months and every 3 months after surgery. The patients were followed -up for postoperative complications, recurrence and mortality. They were examined with ultrasonography, CT scan, MRI and also liver function tests. Diagnosis was made clinically and imaging in five (27.78%) patients which was confirmed on surgery. In 13 remaining (72.22%) patients, the diagnosis was made based on imaging guided Tru-Cut biopsy in five (38.46%) patients or diagnostic laparotomy with mass biopsy in eight (61.54%). 

## Results

A total number of 18 patients were investigated in the study. Fourteen (77.78%) patients were from suburb of Chenaran town, Khorasan Razavi province, Iran; two (11.11%) from Mashhad; one (5.56%) from Kalat, Khorasan Razavi province; and one (5.56%) case was from the neighboring country of Turkmenistan. The mean age was 46.11±15.14 years (median of 47 and range 23-74 years). There was a predominance of women (15, 78.9% females vs 4, 21.1% males) and the difference was significant (P=0.012). The presenting symptoms in all patients except one, were epigastric pain in 16 (88.89%) patients, cholestatic jaundice in three (16.67%) patients, and fever in two (11.11%) patients, while in one (5.56%) patient has cough and bloody sputum, fatigue, and ascites. Only in one (5.56%) case the disease was detected incidentally during laparotomy for excision of uterus fibroma. But the chief complaint was epigastric pain in 14 (77.78%) patients and icterus in 2 (11.11%) patients. 

The mean serum total bilirubin was 2.80±4.80 mg/dl (range from 0.3 to 29.3 mg/dl), the mean serum SGOT, SGPT and alkaline phosphatase level were 37.5±22.17 u/l (18–151 u/l), 39.3±13.67 u/l (10–92 u/l) and 678±303.83 u/l (96–1919 u/l), respectively. The mean symptom duration time of prior to diagnosis and treatment was 26.6±29.83 months (range 1-180 months). Biopsy was helpful only in 3 patients (table 1).

**Table 1:** Procedures performed for the diagnosis of Alveolar Echinococcosis

**Procedures**	**No of cases(%)**	**Accuracy**
Trucut biopsy	5 (27.78%)	60.0% (3 out of 5 cases)
Laparotomy and biopsy	8 (44.44%)	0.0%
Clinical manifestations and imaging	5 (27.78%)	100.0%

At first presentation, the primary diagnosis in 7 patients was hydatid cyst and in 1 patient tuberculosis and overall misdiagnosis was made in 10 patients. The definitive diagnosis in these patients was made after the mean delayed time of 51.2±26.8 months. Therefore, misdiagnosis was made in 8 patients and correct diagnosis in another 8 patients. Various segments of the liver were differently involved. The right lobe and segment IV in five (27.7) cases, left lobe and segments V and 8 in four (22.22%) cases, right lobe in two (11.11%) cases, left lobe in two (11.11%) cases, segment IX and XII in one (5.56%) case, segments IV and V in one (5.56%) case and multiple involvement in both lobes in three (16.67%) cases. 

Different modalities were used for treatment such as liver radical resection in seven (38.89%) patients, biliary bypass in two (11.11%) patients, laparotomy and tumor biopsy in eight (44.44%) patients and medical treatment in one (5.56%) case (table 2). 

**Table 2:** Procedures used for the management of patients with Alveolar Echinococcosis and their outcome

**Procedure**	**No of cases (%)**	**No of deaths (%)**	**Cause of mortality (%)**
Liver resection	7 (38.89)	1 (14.29)	Cholangitis
Palliative surgery (biliary bypass)	2 (11.11)	2 (100.0)	Cholangitis 1 (50.0) Biliopulmonary fistula (respiratory insufficiency) 1(50.0)
Inoperable (diagnostic laparotomy)	8 (44.44)	0 (0.0)	-
Trucut biopsy (medical treatment)	1 (5.56)	1 (100.0)	Lung metastasis
P-value	0.042	--	--

**Figure 1:**
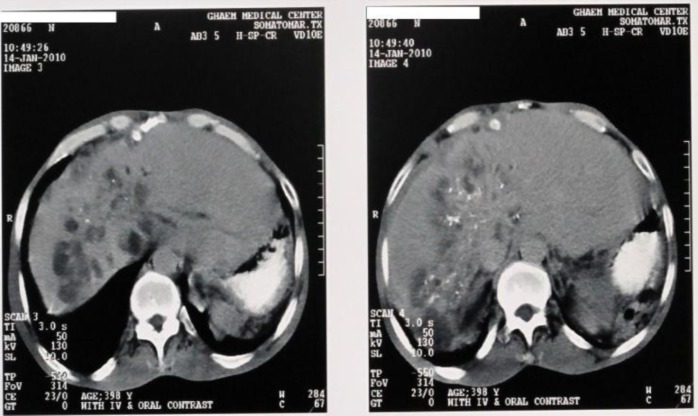
CT scan of the patient suffered from biliopulmonary fistula shows multiple cysts and microcalcifications and plaquelike calcific foci within and around the lesion

**Figure 2:**
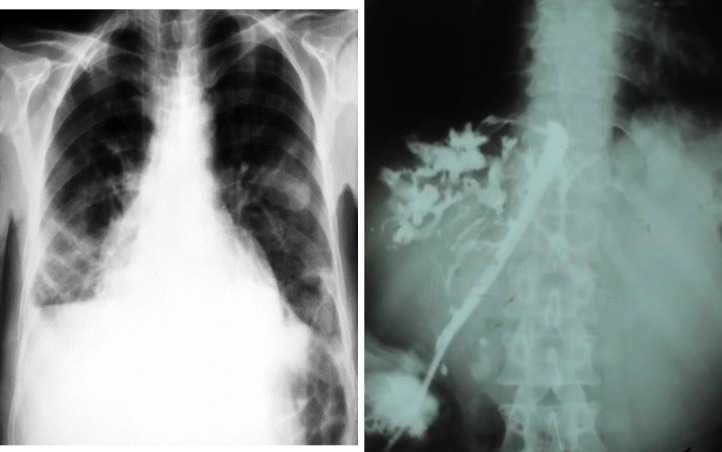
Chest X- ray and fistulography of the same patient with biliopulmonary fistula have shown a right basal consolidation due to direct extension of the liver process to the lung and changes like sclerosing cholangitis

The distribution of the procedures was statistically significant (0.042). Hepaticojejunostomy of the segment III was done in one (5.56%) patient. Also, in one (5.56%) patient with postoperative biliary fistula, the anastomosis of the fistula tract to jejunum was performed. The latter patient, suspicious of having a hydatid cyst, has been operated and died after 32 months due to biliopulmonary fistula (Figure 1, 2).

**Figure 3:**
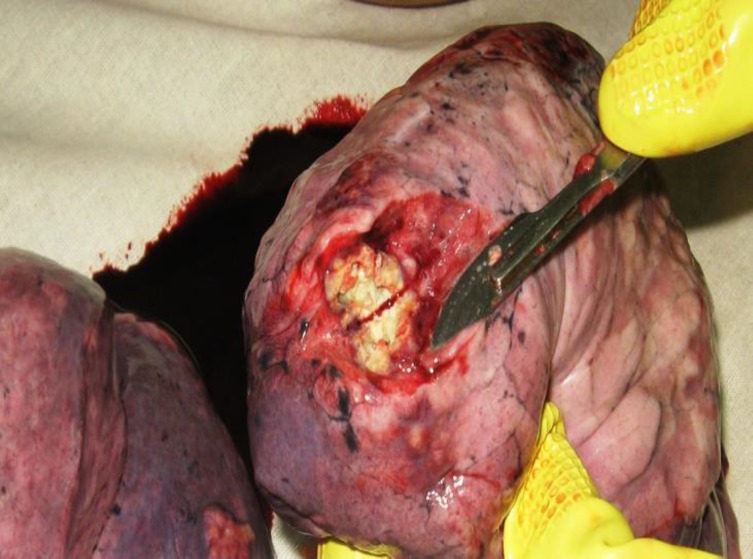
Postmortem specimen of lung parasitic metastases in a 38-year old female patient

The lung was involved in four (22.22%) patients including three parasitic metastases and one direct extension of the liver process (Figure 3). In the follow-up period, four (22.22%) patients were lost from follow-up after mean survival time of 45.70±7.50 (18–63) months (two patients because of post-operative cholangitis, one due to biliopulmonary fistula and one because of lung metastasis). Until now 14 (77.78%) patients are alive with mean follow-up period of 54.60±29.17 (2-177) months. All patients received post-operative chemotherapy for two years after radical surgery and are still under close follow-up. A long-term chemotherapy with benzimidazole derivative was administered for nonresectable cases.

## Discussion

Alveolar echinococcosis was first reported in the mid-19th century. Based on the studies of Vougle (1955) in Germany; Echinococcus granulosus and Echinococcus multilocularis were considered as two distinct species (7). Human alveolar echinococcsis is a progressive and potentially fatal parasitic infection. Early diagnosis of alveolar echinococcosis is difficult because the disease has an asymptomatic period which can last up to 20 years (8). Liver lesions due to alveolar echinococcosis can be different in terms of size from a small focus with few millimeters to large masses with diameter of 15 to 20 cm.

 Clinical diagnosis includes epidemiological data, characteristics of lesions in imaging studies (ultrasound, CT scan, etc.) and serologic markers. Alveolar echinococcosis is characterized by calcification in or around the liver lesions (seen in 70% of cases). In the evaluation of 70 patients with unresectable alveolar echinococcosis, the primary clinical symptoms were epigastric pain (one-third) or obstructive jaundice (one-third) and in other cases the disease was diagnosed accidentally (9). 

In our study, primary symptoms included epigastric pain in 16 cases of 18 patients and jaundice in three patients and only one case was detected accidentally during laparotomy due to gynecologic disease. Metacestode tends to spread through metastasis from liver to other organs. Liver alveolar echinococcosis metastasis is mainly through the bloodstream. Pulmonary metastasis occurred in 20% of patients, while brain metastasis had been reported only in 1% of the cases. Pulmonary alveolar echinococcosis is mainly due to hematogenous spread of AE lesions of the liver. Clinical signs and symptoms in pulmonary AE include hemoptysis, chest pain, sputum in cough and dyspnea during activity (8). In our patients, four (22.2%) out of 18 patients had lung involvement along with hepatic involvement (three cases due to metastasis and one case due to direct expansion of the disease from liver to lung).

A case of a 47-year-old woman with alveolar echinococcsis of the liver and Budd-Chiari syndrome with the presentation of variceal gastrointestinal bleeding has been reported (1). Radical reports. (9) In our study, the rate of complete removal of the lesion was possible in 7 (38%) patients. Since the current treatment with imidazole is only parasitostatic, the patients with inoperable AE require lifelong drug treatment. In some cases of human AE, spontaneous improvement of the disease has been seen attributed to immune response leading to growth suppression parasite in human. These cases are known with calcified parasitic lesions (10).

In AE, human is incidental intermediate host of larva Echinococcus multilocularis, (11) whereas rodents are the most common intermediate host, and carnivores such as foxes are the final hosts of the parasite that cause spread of the disease through their feces (12). Most cases of alveolar echinococcosis have been reported in China (13). In Iran, all previous reported cases of the disease were related to Khorasan Province, North East of Iran (14, 15) and Azerbaijan Province, North West of Iran (16, 17) and in those patients living in rural areas with low levels of health and medical services. 

Other presentations of disease include, fever, right upper quadrant abdominal pain, jaundice, itching, and portal hypertension (18, 19). Early diagnosis is needed to prevent the complications and proper management of patients (20). CT scan is one of the basic diagnostic methods for the diagnosis of AE (21) Although surgical resection or organ-sparing interventions in the early stages of the disease can be considered the best treatment modalities, these surgeries can be done only in 40% of cases (20). According to the guidelines of World Health Organization (WHO), we prescribed drug therapy after radical surgery for two years and control the patients for a minimum of 10 years. Long term drug therapy with benzimidazole derivatives (mainly albendazole) is prescribed for unresectable cases. Long-term treatment with benzimidazole derivatives is generally well-tolerated. To the best of our knowledge, this disease has been reported as sporadic in Iran, but in the past two decades, we found an endemic area with a high incidence of the disease in Chenaran, a town in Khorasan Razavi province.

In conclusion, alveolar echinococcosis is one of the most fatal parasitic infections in human beings that early diagnosis and radical surgery provide the best chance for definitive cure of the patients.
